# Living at the end-of-life: experience of time of patients with cancer

**DOI:** 10.1186/s12904-019-0424-7

**Published:** 2019-05-14

**Authors:** Jordy Johannes Eduardus Rovers, Elze Jantien Knol, Jelte Pieksma, Wytse Nienhuis, Anne Barbara Wichmann, Yvonne Engels

**Affiliations:** Radboud University Honours Academy, Nijmegen, Gelderland The Netherlands

**Keywords:** Palliative care, Time perception, Patients, Qualitative research

## Abstract

**Background:**

The aim of this study was to gain insight into the experience of time of terminal patients with cancer. Experience of time is relevant in palliative care in both policy and practice. On a policy level, the Quality Adjusted Life Year (QALY), the most used outcome measure for cost-effectiveness analysis in healthcare, assumes time to be a linear and additive variable, which is one of the reasons that its applicability in palliative care is questioned. On a practice level, a better understanding of the experience of time of patients with limited time left, could help to recognize if and how these patients can have a more meaningful use of time. The main focus of this study was to discover whether time perception of these patients in their last months of life had changed as compared to earlier periods of time in their lives in good physical health. The pace of time and time dominance (comparison of past, present and future) were investigated.

**Methods:**

In several hospices and palliative care units in the Netherlands, twelve semi-structured interviews were conducted with terminal patients with cancer.

**Results:**

Time perception at the end of life had changed for most participants. They all lived on a day-to-day basis in the terminal phase, independent of their way of life in the healthy phase. Furthermore, the experienced duration of a day turned out to be very different between patients, but also between days, depending on daily activities. Besides, for most patients for whom the future was the dominant period of time in the healthy phase, the dominant period of time in the terminal phase had become the past.

**Conclusions:**

Time perception of terminal patients with cancer differed from the time perception in their relatively healthy phase of life. This suggests that the LY part of the QALY is not comparable for all phases of life.

**Electronic supplementary material:**

The online version of this article (10.1186/s12904-019-0424-7) contains supplementary material, which is available to authorized users.

## Background

One of the earliest documented discussions on the nature and experience of time was by St. Augustine about 1600 years ago [1]. He found that time is impossible to grasp and yet it always exists in the present. Also today the experience of time is debated by philosophers and (neuro)scientists [2]. There is no consensus on what perception of time is and how it is governed in the human brain [3, 4]. This makes time a difficult topic to study.

When studying time perception, clock time and embodied time should be distinguished [5–7]. People are better equipped to perceive changes in numbers, magnitude and prominence of events in a given time interval, then experiencing time as an independent variable, due to a lack of ‘temporal receptors’ [7]. Therefore, experience of time can be defined as the experience of events. Time intervals that contain more stimuli (e.g. motion, visual information) tend to be experienced as if the duration is shorter than time intervals containing fewer stimuli [8–10]. For example, lying awake at night, contemplating, can feel significantly longer than receiving a visit from your grandchildren during the day.

Being diagnosed with a terminal illness disrupts many aspects of life [11, 12], among which the experience of time [13–15]. Lövgren et al. showed that the time experience of patients with inoperable lung cancer tends to change after their diagnosis [15] and van Laarhoven et al. showed that time experience differs between cancer patients with advanced disease and disease-free cancer survivors [16].

Experience of time is relevant in palliative care in both policy and practice. On a policy level it is important to understand the experience of time of patients to make decisions about health interventions. It plays an important role in the Quality Adjusted Life Year (QALY), the most used outcome measure for cost-effectiveness analysis in healthcare [17, 18]. A debate regarding its applicability in palliative care is ongoing [19]. The QALY has two main determinants, the quality of life (Q) and life years (LY) gained by health interventions [20]. In the QALY, time is considered to be linear and additive (clock time). This is one of the reasons for discussions on its applicability for comparing curative health interventions and palliative care interventions and seemingly results in unfavorable financial allocation for palliative care [21]. Moreover, the QALY is a measurement tool, used on a macro level, that should be representative for what is important on a micro (patient) level.

On a practice level, a better understanding of the experience of time of terminally ill patients may help to recognize if and how these patients can have a more meaningful use of time. When a health care professional and patient talk about the diagnosis and prognosis of incurable cancer, they discuss the decreased life expectancy [22], even though this is difficult to estimate correctly [23]. In such discussions, often only clock time is taken into account, while the embodied time is not addressed. It is known that in patients with cancer there is a relation between a focus on the past and distress [16] and that patients in this group have an increased appreciation of aspects of daily life [15]. Health care professional-patient conversations regarding experience of time may help to find daily activities best fitting the patient’s wishes, aiming at making the most of their remaining time. However, insight in differences in experience of time in the terminal phase compared to an earlier healthy phase of life is limited.

In this study, we explored the experience of time of terminally ill patients with cancer in their healthy past and in their current terminal phase.

## Methods

### Study design

Semi-structured interviews were conducted, in which patients introspectively compared their current experience of time with that in a healthy phase of their lives. The interviews were conducted by master students from multiple disciplines. The interviewers explained to each patient that they were interested in the patient’s experiences and views on this topic. All interviewers had interview training and were experienced in conducting interviews. Each interview was conducted by two people, who discussed the interviews afterwards, aiming at accounting for assumption and biases.

**Fig. 1:**
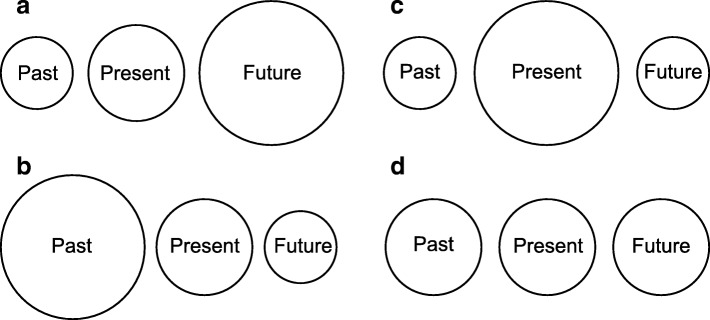
Illustration of some of the possible outcomes of Cottle’s Circles Test. **a** Future dominance **b**) Past dominance **c**) Present dominance **d**) A different outcome: Past, present and future are equally valued

A semi-structured topic list (Table 1) was developed. For each topic, experiences, feelings and emotions were explored through open-ended questions. The first topic - pace of time - was explored solely through open-ended questions. In the second topic - time dominance - patients were firstly asked to compare the past, present and future by performing Cottle’s Circles Test [24]. In this test, patients are asked to draw three circles representing the past, present and future. Time dominance is then assessed by comparing the sizes of the three circles. If the circle representing the past is the largest, past dominance is indicated. In the same manner, past and future dominance are determined. There are also other situations, for example if all circles are equally large (Fig. 1). In this study firstly Cottle’s Circles Test was conducted, which was used as a starting point for a subsequent conversation using open-ended questions regarding time dominance. The results were drawn from the conversations, not from the drawings, thus correcting for patients who changed their minds. For the full interview protocol, see Additional file 1: Appendix 1.

**Table 1:** Topics and subtopics interviews

Topics	Subtopics
Pace of time	Pass of time
	Idea of passing of time in healthy phase
	Idea of passing of time in palliative phase
Time dominance	Cottle's Circles Test
	Main difference between time dominance in the palliative and healthy phase

### Participants

In the Netherlands, patients can be referred to a hospice or a palliative care unit in a nursing home if their life expectancy is three months or less. Via several hospices and palliative care units in the eastern part of the Netherlands, patients with cancer were recruited through purposive sampling: potential participants were identified and approached by their healthcare professionals, who provided them with written and verbal information regarding the content and goal of the study. The in- and exclusion criteria are listed in Table 2. This study was restricted to cancer patients to prevent bias based on the underlying (terminal) disease. The hospices informed our study team in case there were patients meeting the criteria, after which one or two researchers travelled to the hospice to conduct the interviews. Upon arrival, the interviewers discussed briefly with staff members if there were significant changes in the health of the selected patients since they were selected for the interview (within approximately two weeks). When meeting the participating patients in person, the interviewers themselves made the executive decision if the patient’s medical state allowed participation. The interviewers emphasized that no answers were expected if questions were too personal, that the patient could ask questions at any time, and that they were allowed to cancel participation at every moment. The hospices, palliative care units and patients did not receive any payment for their participation.

**Table 2:** Patient eligibility

Inclusion criteria	Age of ≥18 years
	Having a life-threatening oncologic disease with a prognosis of 3 months or less (hospice setting)
	No evidence of cognitive disorders (physicians’ judgement)
	Being able to read and speak Dutch
	Written informed consent
	Being able to reflect on their disease (physicians’ judgement)
	Not receiving anti-tumor therapy and recovered from acute treatment toxicities at the moment of inclusion
Exclusion criteria	Extreme morbidity precluding the ability to speak for 30 min (Karnofsky Performance Scale 40)
	Clinical signs of severe mood disorders (physicians’ judgement)

### Analysis

All interviews were audio recorded and transcribed verbatim and field notes were made. After anonymization, the transcripts were imported in ATLAS.ti (version 7) for data analysis. An inductive iterative process was performed using thematic content analysis [25]. Line by line, systematic content analysis of the first two interviews was performed separately by two researchers. Codes were assigned to the different text segments in the transcripts. The codes were grouped into categories by similarities and these formed themes. After three interviews, the researchers discussed existing codes, the topic list and the need for new codes and/or adaptation of the topic list. New codes were added and used for further analysis. In reporting the findings of this study, the COREQ (COnsolidated criteria for REporting Qualitative research) guideline was used [26].

## Results

Saturation was reached after seven interviews with an average duration of 40 min. In total twelve patients were interviewed. The patients were between 61 and 83 years old. Eight patients were male. One patient did not complete the interview due to fatigue. Patient characteristics are listed in Table 3. In the analysis, two themes (pace of time and time dominance) were identified. The interviews were held between January 2017 and April 2017.

**Table 3:** Patient characteristics (*n* = 12)

No.	Sex	Age	Highest level of education	Setting
1	F	80–85	Primary school	Palliative care unit
2	M	75–80	Secondary vocational education	Palliative care unit
3	F	75–80	Secondary school	Palliative care unit
4	M	65–70	University: economy	Hospice
5	M	80–85	Primary school	Hospice
6	F	60–65	Secondary vocational education	Hospice
7	M	80–83	Primary school	Hospice
8	F	70–75	Secondary vocational education	Hospice
9	M	70–75	Higher vocational education in English language	Hospice
10	M	75–80	University: business administration	Hospice
11	M	80–85	Primary school	Hospice
12	M	80–85	Grammar school	Hospice

### Theme 1: pace of time

Eight out of the twelve patients experienced a different pace of time in the palliative phase as compared to the healthy phase, three patients did not experience a difference and one patient did not give a conclusive answer.

All patients noted they lived on a day-to-day basis in the palliative phase. Some already lived on a day-to-day basis in the healthy phase, while others used to live on a week-to-week or event-to-event basis. The limited life expectancy, resulting in uncertainty about the near future, was mentioned as one of the causes for this change and therefore about the possibility to carry out their plans. This eventually resulted in a mindset of not planning ahead. One patient described this uncertainty as follows:

*“I am feeling pretty good and can keep my head straight. But tomorrow it might just be completely different.”* [Patient no. 7]

Patients also mentioned a sense of losing control because of the physical incapability to undertake the activities they used to do.

When exploring day length in the palliative phase, patients gave various answers, ranging from fast to normal or slow passing of the day. In comparison to the healthy phase, three patients experienced a change in duration.

*“Yes, [when I was physically healthy] time went much faster. The days flew by.”* [Patient no. 5]

Additionally, nine patients noted that duration of a day was different each day, both in the healthy and palliative phase of their lives. Differences in daily activities contributed to this fluctuation. Visits from family and friends for example, gave them the feeling of a faster pace of time.

*“One day goes by quickly and the other goes very slowly. Take yesterday: it was really a long day, but you also know why [there was not much to do]. But today I am here with you and I will receive another friend after you, then time really does fly by.”* [Patient no. 2]

The amount of activities per day could be a marker of a pleasant and quick passing of the day for patients in a hospice.

Although it was not specifically asked, two patients mentioned that the duration of the night was problematic. When they were alone at night, they seemed to ponder a lot. This could be a marker of a less pleasant passing of time.

*“What I do experience is that I don’t dare to sleep without the lights on anymore and the curtains must always be open. […] I sleep poorly due to the contemplating.”* [Patient no. 5]

### Theme 2: time dominance

Eleven patients took part in the Cottle’s Circle Test. The other patient stopped the interview preliminary due to fatigue, but did make a comparison between the palliative and healthy phase on this topic. The results of time dominance in the palliative phase are listed in Table 4.

**Table 4:** Results time dominance in palliative phase

Result time dominance	
Future dominance	3
Present dominance	0
Past dominance	4
Present and future valued equally important, past valued less important	1
Past, present and future valued equally important	3
No answer	1

Patients who sensed their past as most dominant and their future as least dominant, argued this was the case because they had limited time left. They preferred to think about good times from their pasts.

*“About the future, I can obviously not talk about that for very long.”* [Patient no. 7]

The three patients who experienced future dominance in the palliative phase, argued that they had many concerns about their future. They stated that they drew the future circle relatively large, because of the worries they harbored about it. Their thoughts seemed to be occupied with the future, making it a bigger part of their current life. Especially the uncertainty of this future made the patients contemplate about it a great deal.

*“The future is a big circle, because I do not know what the future holds.”* [Patient no. 2]

Other patients also mentioned that they worried about the future. However, this did not necessarily make it the most dominant period of time. One patient phrased his concerns about the future as follows:

*“Of course I know what the future will bring me, that is death. That is what it eventually brings everyone. But I just don’t know how this will be, how…? how…? how…? Is that, will that be terrible or will that not be so bad at all? […] And the future, well, I think it is equally important as the past.”* [Patient no. 9]

Eight patients made a comparison between the palliative and healthy phase. The results are listed in Table 5. The remaining four patients did not compare the palliative and healthy phase on this topic, since they either did not fully understand the question, or did not know how they felt about it.

**Table 5:** Results time dominance, comparison palliative and healthy phase

Result comparison	
No difference in time experience	4
Future was more dominant during healthy phase	4
Past and/or present was more domi-nant during healthy phase	0
No answer	4

Compared to the healthy phase, four patients experienced the future as less important in the palliative phase. These patients stated that they used to have a busy life, being involved in many activities.

*“The future used to be much more important, I was a planner.”* [Patient no. 4]

The other four patients experienced no differences in dominance between the palliative and healthy phase. They experienced each time period as equally important throughout life. One patient had a view on time that corresponds with ‘embodied time’ and linked this to being almost indifferent when it comes to the experience of time. She explained her view on time as follows:

*“It is abstract, time cannot be grasped. One moment you think ‘I still have one hour’ and when you check again it is only five minutes. […] My biorhythm tells me my own time. There is a built-in clock that I possess, which ensures that I always do things at the time they need to be done. I don’t need a watch. That is why time doesn’t mean so much to me.”* [Patient no. 8]

Others mentioned that, in a way, past, present and future are equally important since in all the time periods they have nice memories or plans.

*“I would not know from which time period I would be able to collect anything bad.”* [Patient no. 8]

## Discussion

Changes in experience of time of terminally ill patients with cancer in their current, terminal phase compared to the healthy phase of their lives were explored.

Specifically, the experienced pace of time and time dominance (comparison of past, present and future) were researched. When comparing their experiences to the healthy phase, the majority acknowledged a change in pace of time. The patients were divided regarding pace of time. Independent of their lifestyle in the healthy phase, all patients lived on a day-to-day basis in the palliative phase. These findings are in line with the findings by Lövgren et al. [15].

Regarding time dominance, some patients experienced future dominance, some past dominance and some experienced no dominant time period in the palliative phase. Patients who valued the past most in the palliative phase, found the future most important in the healthy phase. These findings are not in agreement with the findings by van Laarhoven et al., who found that the present time was most dominant in patients with advanced cancer [16]. Van Laarhoven et al. also found that disease-free cancer patients mostly experience future dominance. Combining their and our findings, we hypothesize that in general healthier people are more focused on the future than terminally ill patients.

The results on both pace of time and time dominance indicate that there is a shift in experience of time in the terminal phase in comparison with the healthy phase.

### Challenges in the interviews

The subject of time is difficult to grasp, understand and discuss. This sometimes resulted in confusing or inconsistent answers during the interviews. For example, on the pace of time, one patient initially gave the strong impression that her experience of pace of time was rather consistent, but her following answers suggested that her perception of time had changed since she received palliative care.

Furthermore, there were some challenges in interviewing some of the patients in this group. As mentioned in the method section, the interviewers made the final decision if the recruited patients were able to participate. In two events this led to cancellation of the interview. One patient received deep palliative sedation due to a rapid decline in her physical health and refractory symptoms. Another patient was in a confused and very emotional state, so that the interview was cancelled after the introduction. One of the twelve patients that made the final sample did not finish the interview due to fatigue.

To make a comparison between the terminal and healthy phase, patients were asked if they could recall a phase in their lives in which they were healthy or were able to fully participate in everyday activities. The Peak-End-Rule of Kahneman states that the memory of an event or period can be different than the experience at the time [27]. As a consequence, patients arguably have different ideas about their perception of time in their healthy phase than they did when they were actually healthy.

### Strengths and limitations

To our knowledge, this is the first qualitative study in which experience of time in the terminal and healthy phase was compared. This study adds in-depth knowledge about the change of perception of time. The findings may help to recognize how palliative patients can have a more positive experience of time in the final phase of their lives. E.g., considering the experience of time in health care professional-patient conversations may help to identify the needs of palliative patients concerning their daily routines. Additionally, more insight into the applicability of the QALY for cost-effectiveness analysis in palliative care could be achieved as the QALY assumes time a to be a linear and additive variable, which is questioned by the outcome of our study and others, the QALY may not be applicable.

A limitation of this study is that the duration of nights was not studied in depth. Other research suggests that this matter is a problem for palliative cancer patients [28]. Furthermore, the participants had a small range in age, sex and culture. The youngest patient was 61 years and the oldest was 83. Therefore it is not possible to say anything about changes in time perception of younger palliative patients. It is possible that elderly, regardless of their incurable diseases, have a different time perception than younger people [29]. The majority of the interviewed patients were male. All but one were Dutch. The interview with the patient of foreign origin indicated that cultural differences can play an important role in the well-being and experience of time of palliative patients who live in hospices with a Dutch culture concerning e.g. food and the type of activities. Finally, like in other studies [15, 16], we acknowledge that it is very difficult to measure time. Especially because looking back on a certain time period is difficult when that period is not completed [27].

## Conclusions

Our results suggest that there is a shift in time experience throughout different periods of life. All patients in the terminal phase lived on a day-to-day basis, independent of their lifestyle in the healthy phase. Additionally, patients who experienced the past as dominant in the palliative phase, experienced the future as dominant in the healthy phase.

### Recommendations

In future research, valuation of time could be incorporated into interviews to get a more complete view on the subject. We suggest quantitative follow-up research with questionnaires concerning the same topics as we identified in our research. An interesting addition to these topics would be the experience of duration of a night instead of only that of a day.

On a policy level, the impact of the experience of time on the applicability of the QALY in palliative care needs further exploration. The results of our study suggest that the experience of time in the terminal phase is different from the healthy phase, which raises questions on the equal treatment of the LY-component of the QALY in curative and palliative care.

On a practice level, we recommend that healthcare professionals discuss experience of time with their terminal patients. This might facilitate patient centered changes in daily routines regarding e.g. activities, (family) visits and topics of conversation (e.g. the past, present or future). If patients live on a day-to-day basis, it may make more sense to plan activities for maximum one day ahead. Moreover, if the patient has a clear focus on the past, present or future, this may give clues as to what they would like to talk about. These changes might improve patients’ experience of time and their quality of life.

Finally, the influence of culture and socialization on the perception of time may be valuable information, so we recommend this is further explored in the future.

## Additional file

Additional file 1: Appendix 1. Interview protocol. (DOCX 12 kb)

## Abbreviations

COREQ: COnsolidated criteria for REporting Qualitative research
QALY: Quality-Adjusted Life Year
