# Supplementary material for: Living at the end-of-life: experience of time of patients with cancer
Source: BMC Palliat Care. 2019 May 14;18:40. doi: 10.1186/s12904-019-0424-7 (PMC6518794; doi:10.1186/s12904-019-0424-7)
Supplement: Supplementary file 1 — Appendix 1. Interview protocol. (DOCX 12 kb) [file 12904_2019_424_MOESM1_ESM.docx]

**Appendix I Interview protocol**

Please note: the questions below served as discussion topics and were not ask literally, but were integrated in a semi-structured interview.

**1. Introduction**

We will start this interview by introducing ourselves. We are [name 1] and [name 2] and we would like to ask you –again- if it is possible to conduct an interview with you for our research. As you have read and heard earlier from your doctor, we are here on behalf of the Radboud University Nijmegen. We are conducting a research based on the concept of time. Our goal is to get more insights in your experience of time by asking a few questions. [Name 1] will be the interviewer. We will audio record this conversation so we can analyze the information. We will do this anonymously. [Name 2] takes care of the recording equipment and will take some notes if necessary.

If you would like to take a break or have anything to ask or tell to us, including subjects not directly related to this interview, please let us know. If you want to end this interview prematurely, please feel free to do so. We will then stop immediately. We will give you all the time you need for thinking and answering. Please don’t feel any pressure during this interview: there are no wrong answers. Do you have any questions at this point?

**2. General questions**

I would like to start off with a few general questions.

1. What is the highest level of education you received? What kind of education?

2. Do you have a job (or had)? If so, what was your job?

We shortly spoke with your doctor about your process of being ill; when it started and how it developed. Now, I want to ask you some question about this process and your feeling towards it.

3. Since when did you know you were incurable ill?

**3. Pace of time**

I will ask you a few questions about the concept of time. And I will ask you to compare your answer to your point of reference; that is the point you felt healthy/ you were in perfect health.

4. How long did the day of yesterday felt?, How would you compare this to your point of reference?

5. Some people live on a day-to-day basis or week-to-week or event-to-event. What view is fitting with your life now compared to your point of reference?

6. How fast is life passing you by?, “How would you compare this to your point of reference?, "How do you experience this change?

**4. Time dominance**

During this part of the interview we will ask you a few questions where we will ask you simultaneously to make a drawing. We would like to ask you to draw three circles, each representing the past, the present and the future. The bigger the circle, the more important this period was/is for you.

7) Why did you draw the circle representing the past/present/ future, bigger?

8) Why did you draw the circle representing the past/ present/ future, smaller?

9) Which period of time do you prefer to think of? (the past, present or future)

10) Which period of time is the most important for you? (the past, present or future)

11) When you make a decision, do you think of the past, present or future?

12) How do you think you have drawn these circles at your point of reference?

**5. Ending**

13) How important is the extra time left for you?

14) What did you learn about time?

15) Is there anything you would like to tell us?
